# P-607. Respiratory virus co-detections in pre-kindergarten-12th grade students and staff in Kansas City, Missouri

**DOI:** 10.1093/ofid/ofaf695.820

**Published:** 2026-01-11

**Authors:** Brittney Fritschmann, Jennifer Goldman, Nibha Sagar, Anjana Sasidharan, Dithi Banerjee, Olivia Almendares, Hannah L Kirking, Rangaraj Selvarangan, Jennifer E Schuster, Brian R Lee

**Affiliations:** Children's Mercy Hospital, Kansas City, Missouri; Children's Mercy Hospital, Kansas City, Missouri; Children's Mercy hospital, Kansas City, Missouri; Childrens Mercy Hospital, Missouri, Kansas; Children's Mercy Hospital, Kansas City, Missouri; Centers for Disease Control and Prevention, Atlanta, Georgia; Coronavirus and Other Respiratory Viruses Division, National Center for Immunization and Respiratory Diseases, CDC, Atlanta, GA; Children’s Mercy Hospital, Kansas City, Missouri; Children's Mercy Kansas City, Kansas City, MO; Children's Mercy Kansas City, Kansas City, MO

## Abstract

**Background:**

Although respiratory virus co-detections are well-described in medical settings, less is known about them in community-based settings. We used a school-based respiratory virus surveillance platform to examine differences between single and co-detections of viruses among students and staff.

**Table.**
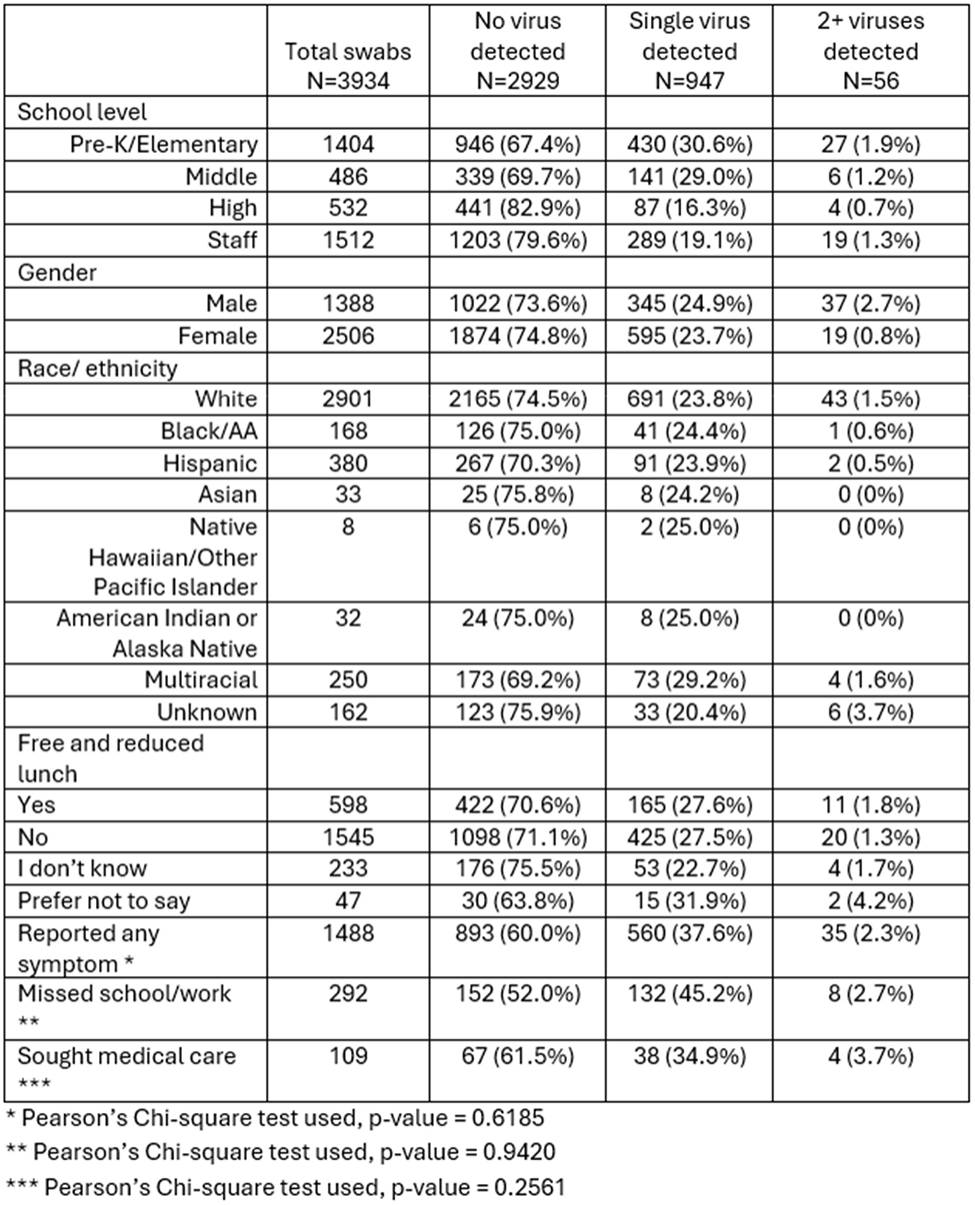
Characteristics of School KIDS participants contributing specimens from August 2024-March 2025

**Figure.**
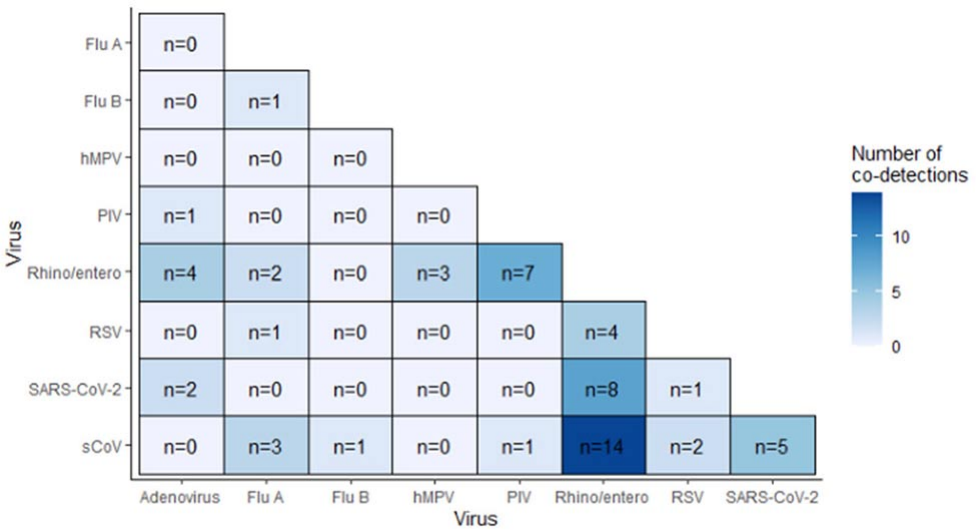
School KIDS Respiratory Virus Co-detection Combinations from September 2024-March 2025

**Methods:**

School KIDS is a longitudinal study assessing respiratory viruses in a pre-kindergarten (pre-K)-12th grade public school district in Kansas City, MO. Self-collected anterior nasal specimens are submitted monthly by participants while at school, and additionally upon request when experiencing ≥1 respiratory symptom, i.e., cough, nasal congestion, fever, shortness of breath, runny nose, sore throat, or wheezing. Specimens are tested via multiplex PCR for adenovirus, rhinovirus/enterovirus (RV/EV), SARS-CoV-2, seasonal coronaviruses 229E, HKU1, NL63, OC43 (sCoV), human metapneumovirus (hMPV), influenza A/B (Flu A/B), parainfluenza virus 1-4 (PIV), and respiratory syncytial virus (RSV). Virus detection was summarized by demographic characteristics and compared across symptom status, absenteeism, and healthcare seeking using Pearson’s chi-square tests. Co-detection was defined as detection of ≥2 viruses.

**Results:**

From August 20, 2024-March 31, 2025, 845 participants submitted 3934 specimens (Table). At least one virus was detected in 1003/3934 (25.5%) specimens, of which 56 (56/1003, 5.6%) had co-detections. Co-detection frequency among positive specimens by school-level were: pre-K/elementary (27/457, 5.9%), middle (6/147, 4.1%), high (4/91, 4.4%), and staff (19/308, 6.2%). The most frequent combinations were RV/EV with sCoV (n=14, 25%), and RV/EV with SARS-CoV-2 (n=8, 14.3%) (Figure). Symptom presence, absenteeism and healthcare seeking did not differ significantly between single-virus detection and co-detection (p >0.05) (Table).

**Conclusion:**

Respiratory virus co-detections occurred in over 5% of positive specimens from students and staff, though clinical outcomes were similar to single-virus detections. Additional work is needed to better understand the role of co-detection in illness severity and transmission dynamics in school settings.

**Disclosures:**

Rangaraj Selvarangan, PhD, Altona: Grant/Research Support|Biomerieux: Advisor/Consultant|Biomerieux: Grant/Research Support|Biomerieux: Honoraria|Cepheid: Grant/Research Support|Hologic: Grant/Research Support|Hologic: Honoraria|Meridian: Grant/Research Support|Qiagen: Grant/Research Support Brian R. Lee, PhD, MPH, Merck: Grant/Research Support

